# The Increase of miR-195-5p Reduces Intestinal Permeability in Ulcerative Colitis, Modulating Tight Junctions’ Expression

**DOI:** 10.3390/ijms23105840

**Published:** 2022-05-23

**Authors:** Viviana Scalavino, Emanuele Piccinno, Giusy Bianco, Nicolò Schena, Raffaele Armentano, Gianluigi Giannelli, Grazia Serino

**Affiliations:** National Institute of Gastroenterology “S. de Bellis”, IRCCS Research Hospital, 70013 Castellana Grotte, Bari, Italy; viviana.scalavino@irccsdebellis.it (V.S.); emanuele.piccinno@irccsdebellis.it (E.P.); giusy.bianco@irccsdebellis.it (G.B.); nicolo.schena@irccsdebellis.it (N.S.); raffaele.armentano@irccsdebellis.it (R.A.); gianluigi.giannelli@irccsdebellis.it (G.G.)

**Keywords:** miRNAs, IBD, intestinal inflammation, intestinal permeability

## Abstract

Defects in the intestinal epithelial barrier functions characterize inflammatory conditions such as Inflammatory Bowel Disease (IBD). Overexpression of pro-inflammatory cytokines such as TNF-α, IL-1B, IL-6 and INF-γ trigger epithelial damage. These cytokines are due to upregulation of claudin-2 (CLDN2) that form a pore channel, resulting in redistribution of TJs and an alteration of barrier permeability. Recently, we demonstrated that miR-195-5p is able to regulate CLDN2 and indirectly also CLDN1 in intestinal epithelial cells. Now, we aimed to investigate the modulation of miR-195-5p on the expression of CLDN2 and other TJs under inflammatory conditions induced by TNF-α. We demonstrated that miR-195-5p also modulated the expression of CLDN2 levels after stimulation with TNF-α. In addition, we discovered the role of miR-195-5p in the integrity of the intestinal barrier and in promoting the restoration of the intestinal epithelial. Moreover, we established that replacement of miR-195-5p attenuated the colonic inflammatory response in DSS-induced, colitis and it reduced colonic permeability. In conclusion, our data revealed the role of miR-195-5p in intestinal inflammation in ulcerative colitis, suggesting a potential pharmacological target for new therapeutic approaches.

## 1. Introduction

The intestinal barrier consists of several elements including intestinal epithelial cells (iECs), the mucus layer, and immunological cells that acts as physical and immunological defense barriers [1,2,3,4]. The epithelial cells form a continuous and a polarized monolayer that acts as a barrier between lumen and the lamina propria, controlling the movements of water, ions, and small molecules through the epithelium. This main function is regulated by junctional complexes located between the membranes of adjacent epithelial cells [4]. The three most important junctional complexes are tight junctions (TJs), adherens junctions (AJs), and desmosomes [5].

An intact intestinal barrier prevents the permeation of pathogens, antigens, and other pro-inflammatory substances [4]. Dysfunction of the intestinal barrier is able to increase intestinal permeability, and it may trigger inflammation and disease. Previous evidence has shown that the TJs play a key role as regulators of the epithelial barrier [6]. The TJs are protein complexes that are composed of members of the claudins family, occludin, and zonula occludens that regulate paracellular permeability, controlling the passage of ions and water through the epithelial barrier, and they guide the correct polarization of epithelial cells [4,6,7].

Defects in the intestinal epithelial barrier functions lead to inflammatory conditions such as Inflammatory Bowel Disease (IBD) [8]. Ulcerative Colitis (UC) and Crohn’s Disease (DC) are the two main disorders included in IBD distinguished by the location of the inflammation in the gastrointestinal tract [9,10]. The increase of pro-inflammatory cytokines with the consequent activation of immunological response lead to the onset of inflammatory diseases in the gut. Several pro-inflammatory cytokines, such as tumor necrosis factor alpha (TNF-α), interleukins (IL-) 1β, IL-6, IL-13, and interferon gamma (INF-γ) contribute to the pathogenesis of IBD. Their dysregulated productions increased the TJ permeability as a result of the loss of the epithelial barrier function, inducing epithelial damage [11,12,13,14,15,16]. TNF-α and IL-1β as well as INF-γ were the pro-inflammatory cytokines with the most elevated concentration in the intestinal tissues of patients affected by IBD [11,17]. It was reported that these cytokines impaired the correct assessment and the functions of TJs in the epithelial barrier [18,19]. Thus, all these pro-inflammatory cytokines may have a key role in the increase of paracellular permeability in intestinal epithelial cells through the redistribution and the expression of TJ proteins [11].

Under inflamed conditions, the deregulated increase of cytokines lead to upregulation of claudin-2 (CLDN2), thus a redistribution of other components of TJs. These alterations trigger a barrier dysfunction that raise its permeability [6,15].

MicroRNAs (miRNAs) are single stranded noncoding RNA able to bind the messenger RNAs (mRNAs), reducing and/or inhibiting protein expression. miRNAs play an important role in regulation of inflammatory responses both in physiological and pathological conditions [20,21,22,23,24].

Several miRNAs that impaired intestinal permeability through the alteration of TJs protein expression have been identified. miR-24 was overexpressed in the colon biopsies of UC patients. Further analysis carried out by Soroosh and co-workers demonstrated that the increase of miR-24 affected the barrier integrity, decreasing cingulin protein expression, which was negatively correlated with UC severity [25]. Wang and colleagues found that miR-223 was upregulated in IBD acting as mediator of CLDN8 expression via the IL-23 pathway. They demonstrated that the inhibition of miR-223 reactivated CLDN8, ameliorating intestinal barrier damage [26]. TNF-α stimulation could influence the expression of miRNAs. In intestinal epithelial cell lines, miR-122a was able to regulate occludin expression with a consequent increasing of TJs intestinal permeability [27]. In the inflamed tissues of IBD patients, miR-200b resulted in downregulation. Moreover, as reported by Shen and coworkers, the increase of intracellular levels of miR-200b ameliorated the TJs injury reducing the expression of IL-8 induced by TNF-α and regulating the c-Jun and MLCK pathways. This ensured the maintenance of the barrier integrity of intestinal TJs [28].

In our recent work, we demonstrated that miR-195-5p was able to maintain barrier integrity, regulating the TJs expression [29]. In the present study, we aimed to investigate whether miR-195-5p modulates the expression of CLDN2 and the other TJs protein expression also under inflammatory conditions induced by TNF-α stimulation. We, firstly, demonstrated that miR-195-5p regulated the expression of TJs even in inflammatory conditions triggered by TNF-α. Then, we functionally characterized in vitro and in vivo the role of miR-195-5p in the regulation of intestinal permeability. These finding supported the basis for a miRNA-based therapy in IBD.

## 2. Results

### 2.1. Effect of miR-195-5p Increase after TNF-α Stimulation

**Figure 1:**
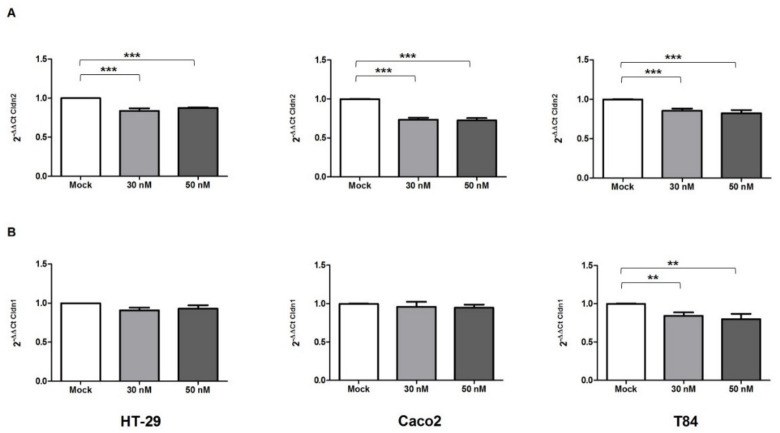
Cldn2 and Cldn1 mRNA expression after miR-195-5p mimic transfection and TNF-α stimulation. (**A**) Cldn2 mRNA expression was analyzed by Real-Time PCR after transfection with miR-195-5p mimic in TNF-α-stimulated HT-29, Caco2 and T84 human cell lines. The increase of intracellular amount of miR-195-5p (at 30 nM and 50 nM concentrations) led to a significant decrease of Cldn2 in all cell lines. (**B**) Cldn1 mRNA expression was analyzed by Real-Time PCR after transfection with miR-195-5p mimic in HT-29, Caco2 and T84 human cell lines. Cldn1 mRNA levels were slightly modulated after transfection but not in a significant manner except for T84 cell line. Mock indicates mock-transfected cells going through the transfection processes without addition of mimic miRNA. Expression data were normalized on the housekeeping gene Gapdh. Data are representative of four independent experiments (mean ± SEM). ** *p* < 0.001; *** *p* < 0.0001.

TNF-α, the most potent pathogenic cytokine in IBD, has a relevant role in intestinal barrier dysfunction mediated by TJ [30,31]. Since TNF-α affect the integrity and the barrier functions of TJ, to simulate the inflammatory condition in vitro, we exposed the HT-29, Caco2, and T84 cell lines to TNF-α treatment, and we evaluated the effect of miR-195-5p on the intestinal epithelial TJ barrier function. We found that transient transfection of the miR-195-5p mimic at 30 nM and 50 nM significantly reduced the Cldn2 gene expression (*p* < 0.05; Figure 1A). Instead, Cldn1 mRNA levels were slightly modulated after transfection but not significantly, except for the T84 cell line (*p* > 0.05 for HT-29 and Caco2 and *p* < 0.001 for T84; Figure 1B).

### 2.2. miR-195-5p Modulates the Structural Organization of TJ Altered by TNF-α

**Figure 2:**
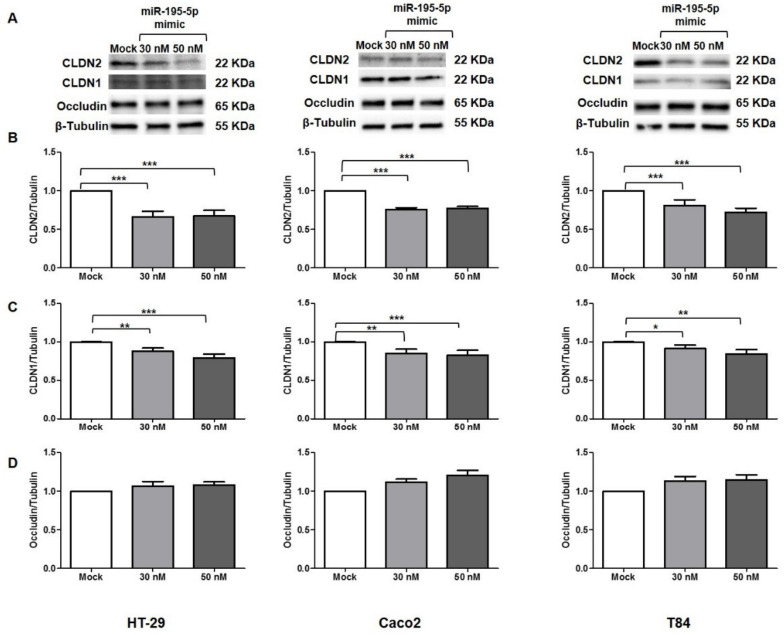
miR-195-5p modulates TJ protein expression after TNF-α stimulation. (**A**) Protein expression of CLDN2, CLDN1 and Occludin following TNF-α stimulation (48 h) in HT-29, Caco2 and T84 cell lines after miR-195-5p mimic transfection. Western blot quantification of CLDN2 (**B**), CLDN1 (**C**) and Occludin (**D**) protein expression in HT-29, Caco2 and T84 cell lines following TNF-α stimulation and after miR-195-5p mimic transfection. A significant reduction of CLDN2 and CLDN1 expression was detected in all cell lines. Occludin protein expression was slightly increased, even if not statistically significantly. Data were obtained by dividing the normalized transfected sample values by the normalized mock-control sample values. β-Tubulin was used as housekeeping protein to normalize the data. Raw data of the independent experiments of Western blot were reported in Appendix A. Data are representative of four independent experiments. The histograms correspond to mean ± SEM. * *p* < 0.05; ** *p* < 0.01; *** *p* < 0.001.

To further confirm miR-195-5p as a regulator of CLDN2 protein expression in inflammatory conditions, we performed transient transfection with miR-195-5p mimic in HT-29, Caco2, and T84 cell lines after TNF-α stimulation. Under this condition, the increase of intracellular miR-195-5p levels resulted in a decreased protein expression of CLDN2 (*p* < 0.05; Figure 2A–C) and CLDN1 (*p* < 0.05; Figure 2A–C) in all three cell lines. In addition, even if not statistically significant, our experimental data demonstrated a slight increase of Occludin protein, indicating that indirect modulation by miRNA could prevent its internalization (*p* > 0.05; Figure 2A–D).

**Figure 3:**
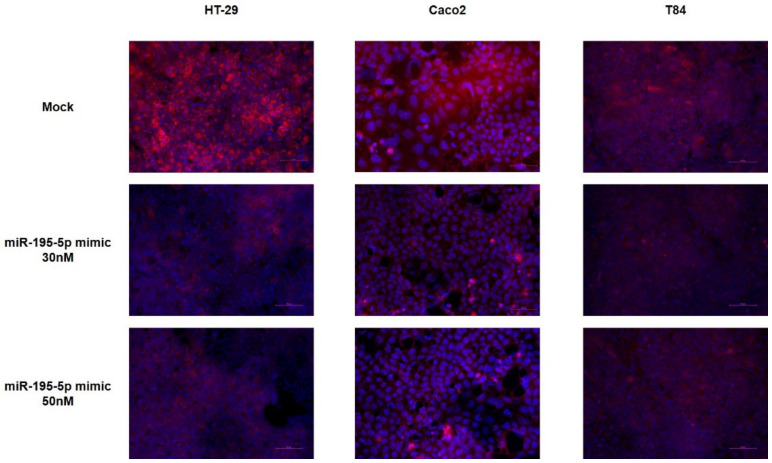
Immunofluorescence staining of cell monolayers exposed to TNF-α and transfected with miR-195-5p mimic at 30 and 50 nM. The immunofluorescence staining shows the same protein expression pattern of western blot analysis. The images were acquired at 20× magnification. Scale bar represents 50 μm.

Furthermore, the immunofluorescence staining in cell monolayers exposed to TNF-α demonstrated that the CLDN2 signal detected in cell lines transfected with miR-195-5p mimic is greatly reduced compared to the mock-control (Figure 3).

### 2.3. miR-195-5p Influences the Intestinal Barrier Function

The expression of pore channels formed by CLDN2 leads to an increased paracellular permeability [31]. To evaluate the integrity and the permeability of the cell monolayer after miR-195-5p mimic transfection, we determined trans-epithelial resistance through TEER measurement (an index to detect the tightness of colonic intercellular connections) in intact and in inflamed HT-29, Caco2, and T84 monolayers.

**Figure 4:**
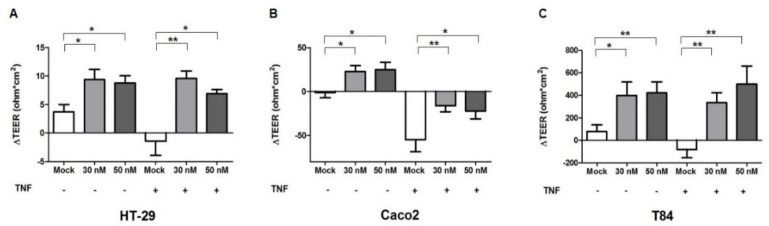
Effect of miR-195-5p in the regulation of intestinal barrier function. Ectopic expression of miR-195-5p in HT-29 (**A**), Caco2 (**B**) and T84 (**C**) cell lines increased the TEER even after stimulation with TNF-α. The value of ΔTEER (ohms*cm^2^) was obtained by subtracting the measured value at 48 h after TNF-α stimulation from the TEER value measured just before the transfection. Data are representative of four independent experiments. The histograms correspond to mean ± SEM. * *p* < 0.05; ** *p* < 0.01.

Our data demonstrated that after raising the amount of intracellular miR-195-5p, the permeability of intestinal epithelial cells as TEER was significantly reduced in all cell lines (*p* < 0.05; Figure 4A–C). Moreover, even after TNF-α stimulation, the TEER of intestinal epithelial cells was significantly enhanced in all cell lines in transfected conditions compared to mock-controls, demonstrating a reduction of intestinal permeability (*p* < 0.05; Figure 4A–C). These results confirmed the role of miR-195-5p in promoting the restoration of the intestinal epithelial.

**Figure 5:**
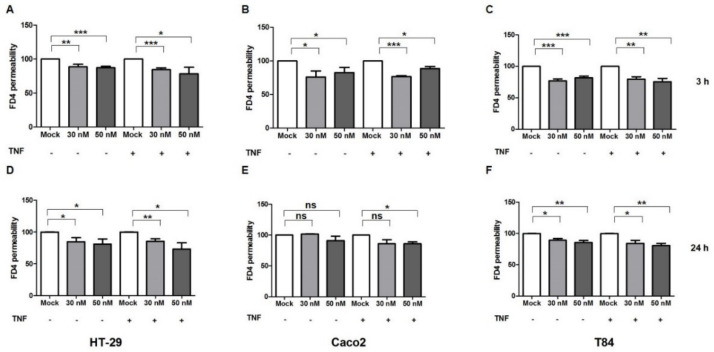
miR-195-5p regulates in vitro intestinal permeability. An increased dextran flux was observed after miR-195-5p mimic transfection in the HT-29, Caco2 and T84 cell lines with and without TNF-α stimulation at 3 h (**A**–**C**) and 24 h (**D**–**F**). Data are representative of four independent experiments. The histograms correspond to mean ± SEM. * *p* < 0.05; ** *p* < 0.01; *** *p* < 0.001. ns: not significant.

To further confirm the role of miR-195-5p in the intestinal barrier integrity, we investigated the effect of an miR-195-5p increase in paracellular permeability through the analysis of a FITC–dextran-4 (FD-4, 4 KDa) assay. FD-4 flux showed a significant reduction in the transfected conditions in all cell lines, considering the mock-control as the maximum amount of permeability (100%) at 3 and 24 h of FD-4 incubation. The reduction was observed also in TNF-α treated cell lines (*p* < 0.05, Figure 5A–F).

These results show that miR-195-5p could control the intestinal epithelial barrier function by targeting CLDN2.

### 2.4. miR-195-5p Attenuates the Intestinal Inflammatory Response in DSS-Induced Colitis

**Figure 6:**
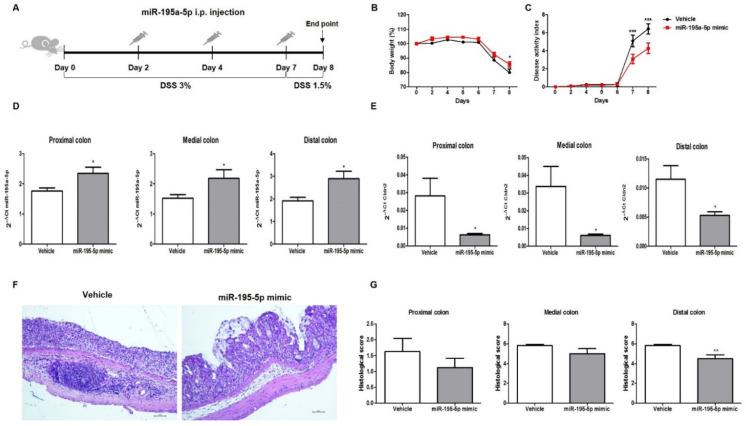
miR-195-5p mimic treatment reduces the intestinal inflammatory response in DSS-induced colitis. (**A**) Schematic diagram of DSS-induced colitis and miR-195-5p administration (n = 8 mice/group). After DSS treatment of mice, body weight loss (**B**) and disease activity index (**C**) were assessed during treatment in each group. (**D**) miR-195-5p expression levels by Real-time PCR in all colon portions (proximal, medial and distal) of treated mice compared to vehicle mice. (**E**) Cldn2 expression levels by Real-time PCR in all colon portions (proximal, medial and distal) of treated mice compared to vehicle mice. (**F**) Representative images of hematoxylin and eosin staining of distal colon tissue from each group taken at 10× magnification. Scale bar 10 μm. (**G**) Histological score of treated and control mice in all colon portions (proximal, medial and distal). * *p* < 0.05, ** *p* < 0.001.

In order to investigate the potential therapeutic action of miR-195-5p in vivo, we employed a DSS-induced colitis model in mice that is an experimental model of human colonic inflammation (Figure 6A).

miR-195-5p administration significantly ameliorated the weight loss induced by DSS in comparison to the vehicle group (Figure 6B). In addition, treatment with miR-195-5p markedly reduced the disease activity index (DAI) score from day 7 onward (n = 8/group; *p* < 0.0001; Figure 6C).

After intraperitoneal administration of the miR-195-5p mimic, we observed an increasing expression of miR-195-5p in all colon portions (proximal, medial, and distal) of treated mice compared to vehicle mice (*p* < 0.05; Figure 6D). According to our in vitro experimental results, CLDN2 expression in colon tissue was strongly reduced in the mice treated with the miR-195-5p mimic compared with the vehicle after the induction of colitis (*p* < 0.05; Figure 6E).

The pathophysiologic structure of the colon was analyzed by H&E staining. Histological analysis of the colon tissue after DSS treatment reveals a destruction of the crypt structure with loss of goblet cells, a disorganized epithelial layer, and massive infiltration of inflammatory cells, while miR-195-5p administration alleviated the colon damage observed in DSS-treated mice (Figure 6F). Considering each portion of the colon, the major difference in terms of a histological score between miR-195-5p-treated mice compared to the vehicle was observed in the distal colon (*p* < 0.05, Figure 6G). This difference was also observed in proximal and medial portions, but it was not significant (Figure 6G).

**Figure 7:**
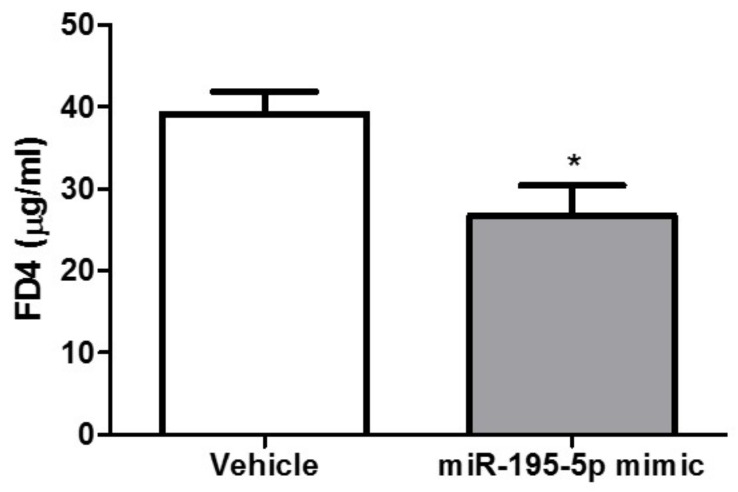
miR-195-5p mimic treatment reduces the in vivo intestinal permeability in DSS-induced colitis. In vivo permeability for FITC-dextran in two independent groups of vehicle and treated mice (n = 5 mice/group). * *p* < 0.05.

Furthermore, in two independent groups of vehicle and treated mice, we found that the in vivo permeability for FITC-dextran resulted significantly reduced (n = 5/group, *p* < 0.05, Figure 7).

Hence, our results demonstrated that miR-195-5p is involved in colon permeability and that increasing miR-195-5p could attenuate colonic inflammation induced by DSS in mice.

## 3. Discussion

The pro-inflammatory cytokines have a relevant role in intestinal barrier dysfunction mediated by TJs [30,31]. TNF-α is a cytokine normally present as a regulator of immune response. An abnormal production of TNF-α plays a key role in IBD and other chronic inflammatory conditions [32,33]. Increased intestinal levels of these pro-inflammatory cytokines were reported in patients affected by IBD [30,34]. In addition, it has a relevant role in intestinal barrier dysfunction mediated by TJs [30].

The exposure of cells by TNF-α lead to an enhanced recruitment of CLDN2 into the tight-junction domain [6,35,36]. Moreover, TNF-α increases the monolayer permeability with consequent alteration of TJs and a decrease in occludin protein expression [13,37,38].

In our recent paper, starting from a miRNA expression profiling of intestinal epithelial cells of an ulcerative colitis mice model, we demonstrated that miR-195-5p resulted downregulated in ulcerative colitis. Moreover, we proved that the intracellular increase of miR-195-5p in colonic epithelial cells led to a decrease of CLDN2 and indirectly, also CLDN1 [29].

Here, we proved that under inflammatory conditions induced by TNF-α stimulation, miR-195-5p was also able to reduce CLDN2 and CLDN1. In addition, even if not statistically significantly, our experimental data report slightly increased Occludin protein levels, suggesting that the indirect modulation by miRNA could prevent its internalization. CLDN2 forms cation-selective pore channels that increase the paracellular flux causing a decrease of transepithelial resistance [39]. We showed that an increase of miR-195-5p corresponds to a modulation of the intestinal permeability of tested cell lines, in terms of TEER and dextran flux.

TNF-α causes an alteration of the TJ-mediated barrier and intestinal permeability, as well as inducing IECs apoptosis [40,41] that precludes the redistribution of TJ and prevents the sealing of the left gaps. Anti-TNF-α, an antibody commonly used in IBD therapy, can ameliorate intestinal permeability but their efficacy depends on the patient’s response [42]. In this context, new therapeutic strategies are needed. Our data suggest that miR-195-5p, even after TNF-α administration, is able to inhibit increased intestinal permeability, monitoring the expression of CLDN2-formed pore channels.

The DSS-induced murine model of colitis is one of the models most widely used to evaluate drug action in UC, as well as to study molecular changes and pathogenesis [43]. To assess the in vivo effect of miR-195-5p on intestinal dysfunction of the colonic barrier, we also used a DSS-mouse model, treated with molecules of miR-195-5p mimic. We found that in vivo the gain-of-function of miR-195-5p also strongly reduced Cldn2 expression, and it distinctively alleviated colon damage in DSS-treated mice. In addition, miR-195-5p-treated mice showed a decreased intestinal permeability, as shown by dextran tracer.

Altogether, our results give further evidence that miR-195-5p regulates the TJ expression in the colon of IBD patients and its dysfunction could be at the basis of the epithelial barrier damage in the disease.

Although our in vitro and in vivo studies have established the involvement of miR-195-5p in the regulation intestinal permeability in IBD, further investigations are needed to confirm its role in chronic ulcerative mice models. Moreover, in this work, we have demonstrated that miR-195-5p are able to restore the intestinal epithelial barrier without considering the changes in the gut microbiota composition. Gut microbiota together with the epithelial intestinal barrier are also considered a fundamental player of IBD. Gut microbiota can also impair the blood brain barrier’s integrity, influencing the trafficking and the secretion of macromolecules and neuroinflammatory components [44,45]. Future studies will be carried out to test the role of miR-195-5p in the homeostasis of gut microbiota and the consequent regulation in the permeability of the blood–brain barrier through the microbiota–gut–brain axis.

In conclusion, in this paper, we demonstrated that miR-195-5p under inflammatory conditions, induced by TNF-α stimulation, modulates CLDN2 and also other components of TJs. In turn, this regulation led to an amelioration in the permeability of the cell monolayer, in terms of TEER and dextran flux. Moreover, in vivo, we clearly proved the role of this miRNA in the integrity of the barrier, as well as a protective role in experimental acute colitis. Our findings confirm the implications of miR-195-5p in the regulation of TJs, typically dysregulated in patients affected by IBD. These results potentially give evidence on the use of miR-195-5p mimic as a powerful therapeutic agent for the treatment of IBD.

## 4. Materials and Methods

### 4.1. Animal Experiments

The animal experiments were conducted according to ethical standards and in accordance with national and international guidelines. The study was approved by the authors’ institutional review board (Organism for Animal Wellbeing—OPBA).

All animal experiments were carried out in accordance with Directive 86/609 EEC required by Italian D.L. n. 26/2014 and approved by the Committee on the Ethics of Animal Experiments of the Ministero della Salute—Direzione Generale Sanità Animale (Authorization n. 337/2019-PR) and the official RBM veterinarian. If in severe clinical conditions, animals were sacrificed to avoid suffering.

### 4.2. Cell Cultures and In Vitro Transfection

For the in vitro experiments, three human colon cell lines were used. HT-29, Caco2 and T84 cell lines were purchased from ATCC (American Type Culture Collection, Manassas, VA, USA). HT-29 and Caco2 cell lines were grown in a culture medium composed of Dulbecco’s Modified Eagle Medium (DMEM, Thermo Fisher Scientific, Waltham, MA, USA) with 10% heat-inactivated Fetal Bovine Serum (FBS, Thermo Fisher Scientific, Waltham, MA, USA), 10 mM HEPES (Sigma-Aldrich, St. Louis, MO, USA), 1 mM sodium pyruvate (Sigma-Aldrich, St. Louis, MO, USA), and 1% streptomycin/penicillin (Thermo Fisher Scientific, Waltham, MA, USA). The T84 cell line was grown in Dulbecco’s Modified Eagle Medium: Nutrient Mixture F-12 (DMEM:F12, Thermo Fisher Scientific, Waltham, MA, USA) supplemented with 10% FBS (Thermo Fisher Scientific, Waltham, MA, USA) and 1% streptomycin/penicillin (Thermo Fisher Scientific, Waltham, MA, USA). All cell lines were maintained at 37 °C in a 5% of CO_2_.

All cell lines were seeded in 12 mm and 6.5 mm transwells (0.4μm) (Corning, Corning, NY, USA) and then transfected with synthetic molecules of miR-195-5p mimic at concentrations of 30 nM and 50 nM (Life Technologies, Carlsbad, CA, USA) using TKO transfection reagent (Mirus Bio LLC, Madison, WI, USA) as previously described [20]. After transfection, cell cultures were basolaterally stimulated with 10 ng/mL TNF-α (PeproTech, Rocky Hill, NJ, USA).

In all transfection experiments, we included a mock control in which cells were transfected with transfection reagent without miRNA mimic.

### 4.3. RNA Extraction and Real-Time PCR

Total RNA, including small RNA, was extracted from cell cultures 24 h after transfection using TRIzol reagent (Invitrogen by Thermo Fisher scientific, Waltham, MA, USA), according to the manufacturer’s protocol. The RNA concentration was determined with the NanoDrop ND-2000 Spectrophotometer (Thermo Fisher Scientific, Waltham, MA, USA).

Total RNA was reverse transcribed using the iScript Reverse Transcription Supermix (BioRad Laboratories, CA, USA), according to the manufacturer’s recommendations.

Quantitative real-time PCR amplification reactions were performed in 20 μL of final volume on a CFX96 System (Biorad Laboratories, Hercules, CA, USA) using the SsoAdvanced Universal SYBR Green Supermix (BioRad Laboratories, Hercules, CA, USA) and the primers PrimePCR SYBR green Assay for Claudin 2 (BioRad Laboratories, Hercules, CA, USA) and QuantiTect Primer Assay for Claudin 1 and Gapdh (Qiagen, Hilden, Germany). Details of used primers are summarized in Table S1. Gapdh gene amplification was used as a reference standard to normalize the relative expression of Claudin 2 and Claudin 1.

For miR-195a-5p detection, total RNA, including miRNA, was reverse transcribed using a TaqMan Advanced miRNA cDNA Synthesis Kit (Thermo Fisher Scientific, MA, USA), following the manufacturer’s instructions. Real-time RT-PCR for the quantification of miR-195-5p was performed using TaqMan Advanced miRNA assays and a TaqFast Advanced Master mix (Thermo Fisher Scientific, MA, USA). miR-186-5p was used as endogenous control to perform the normalization.

Comparative real-time PCR was performed in triplicate including no-template controls.

The relative expression was calculated using the 2^−ΔCt^ and the 2^−ΔΔCt^ formula.

### 4.4. Western Blot

Forty-eight hours after transfection, total proteins were extracted using T-PER Tissue Protein Extraction Reagent (Thermo Fisher Scientific, Waltham, MA, USA) supplemented with cocktail proteinase inhibitors (Sigma-Aldrich, St. Louis, MO, USA). Total protein concentration was determined with the Bradford colorimetric assay (Bio-Rad Laboratories, Richmond, CA, USA).

For each sample, equivalent amounts of proteins were separated on 4–20% Mini-PROTEAN TGX Stain-Free Protein Gels (Biorad Laboratories, Hercules, CA, USA) and then electrotransferred on PVDF membranes (Biorad Laboratories, Hercules, CA, USA). For protein detection, PVDFs were incubated in an automated iBind Flex Western Device (Thermo Fisher Scientific, Waltham, MA, USA) according to the manufacturer’s protocol. Primary antibodies included mouse monoclonal Claudin-1 (sc-166338, Santa Cruz Biotechnology, Inc., Heidelberg, Germany, dilution 1:1000), rabbit monoclonal Claudin-2 (#48120, Cell Signaling, Technology, Danvers, MA, USA, dilution 1:1000), rabbit mono-clonal Occludin (#91131, Cell Signaling, Technology, Danvers, MA, USA, dilution 1:1000), and mouse mAb Tubulin (sc-166729, Santa Cruz Biotechnology, Inc., Heidelberg, Germany, dilution 1:1000). Secondary antibodies included Goat Anti-mouse IgG-(H + L)-HRP conjugate (170-6516, Biorad Laboratories, CA, USA, dilution 1:500) and Goat Anti-rabbit IgG-(H + L)-HRP conjugate (31466, Invitrogen, Carlsbad, CA, USA, dilution 1:2500).

Signal intensities were detected using a Chemidoc System (Biorad Laboratories, Hercules, CA, USA), analyzed with Image lab Software (Biorad Laboratories, Hercules, CA, USA) and quantified by ImageJ Software. The β-Tubulin protein expression was used to normalize the target protein signal.

### 4.5. Immunofluorescence

HT-29, Caco2, and T84 cells were seeded in 6.5 mm transwells (0.4 μm) (Corning, Corning, NY, USA). After transfection and stimulation with TNF-α, cells were washed with PBS and fixed with cold met-OH for 10 min at 4 °C and subsequently. Permeabilization was performed incubating the monolayer with Triton-X 0.1% in PBS for 5 min at room temperature. Samples were blocked in PBS + BSA 3% for 1.5 h at room temperature. Then, samples were stained with primary antibody rabbit polyclonal Claudin-2 (51-6100, Life Technologies, Carlsbad, CA, USA, dilution 1:100) and diluted in PBS + BSA 3% for 3 h. After washing with PBS, they were stained with secondary antibody chicken anti-Rabbit IgG (H + L) Alexa Fluor 594 (A-21442, Invitrogen, Carlsbad, CA, USA, dilution 1:400) in PBS + BSA 3% for 1 h. ProLong Gold Antifade Mountant with DAPI (Thermo Fisher Scientific, Waltham, MA, USA) was applied to each sample and then mounted with a glass cover slip. The fluorescence was observed using an Eclipse Ti2 Nikon microscope (Nikon Inc., Melville, NY, USA).

### 4.6. Permeability Assay

HT-29, Caco2 and T84 cells were cultured on 6.5 mm transwell inserts (0.4 µm) until confluence. The Millicell^®^ ERS-2 Voltohmmeter (Merck Millipore) was used to measure the trans-epithelial electrical resistance (TEER) across the cell layer. The TEER value was obtained by multiplying the resistance value (Ω) to the insert area (cm^2^). After reaching a baseline resistance, cells were transfected as previously described. TEER was measured before and after transfection, and following TNF-α stimulation. Each TEER value was normalized by subtracting the blank, that is an insert without cells.

For permeability assays, HT-29, Caco2, and T84 cell lines were transfected with miR-195-5p mimic molecules at 30 nM and 50 nM and then exposed to TNF-α (10 ng/mL) for 48 h. Each insert was then washed once and balanced for 30 min at 37 °C, 5% CO_2_ with HBSS 1X. Subsequently, HBSS 1X was replaced with fresh HBSS 1X containing 1 mg/mL of 4 kDa fluorescein isothiocynate-conjugated (FITC) dextran was added in the apical portion of the insert and incubated for 3 and 24 h at 37 °C, 5% CO_2_. In each experiment, we included a positive control consisting of a cell monolayer treated with 2 mM EGTA, a molecule that breaks cell junctions. The basolateral media were collected and the FITC concentration was determined using a fluorescent SPECTROstar Omega microplate reader (BMG Labtech) (excitation and emission spectra were 492 nm and 520 nm, respectively).

### 4.7. DSS-Induce Mice Colitis Models and In Vivo Transfection

In the in vivo model, acute colitis was induced by feeding male C57BL/6 mice (8–12 weeks old) with 3% (*w*/*v*) of dextran sulfate sodium (DSS, MW 40,000; Alfa Aesar, Ward Hill, MA, USA) continuously for 7 days in drinking water, then reduced to 1.5% until sacrifice (day 8). Mice were subdivided into two groups: vehicle (group = 8) and treated (group = 8). For in vivo transfection, miR-195-5p mimic (0.5 nmol/mice) was previously complexed with Invivofectamine 3.0 Reagent (Thermo Fisher Scientific, Waltham, MA, USA) according to the manufacturer’s instructions. For the vehicle group, the volume of miRNA mimic was replaced with an equal volume of PBS. Complexes were inoculated via peritoneal injection three times, on days 3, 5, and 7. Body weight, rectal bleeding, and stool consistency were monitored daily after DSS administration. To determine the disease activity index (DAI) score for all animals, a sum of these three values was done [46,47]. For each parameter a score from 0 to 4 was attributed, resulting in the total DAI score ranging from 0 (unaffected) to 12 (severe colitis). Twenty-four hours after the last injection, mice were sacrificed. During sacrifice, colon weight and length were examined, and the colon portions (proximal, medial, and distal) were collected for RNA extraction and histopathological analysis. Total RNA, including small RNA, from tissues was extracted using TRIzol reagent (Invitrogen, Carlsbad, CA, USA), according to the manufacturer’s protocol.

The permeability tracer 4kda FITC-Dextran (Sigma) was also used to perform the intestinal permeability assay using 5 DSS-treated animals/group with and without miR-195-5p mimic administration. Briefly, mice were fasting for 3 h and then oral gavage was performed with FITC-dextran at a concentration of 0.4 mg/g body weight. Four hours after gavage, mice were anesthetized, blood was collected and centrifuged at 3000× *g* for 10 min, and serum was collected. The Fluorescence of FITC-dextran in serum was measured on a FLUOstar OPTIMA Microplate Reader (BMG Labtech) at 490 nm excitation and 520 nm emission wavelengths. The FITC-dextran concentration was determined from a standard curve generated by serial dilutions of FITC-dextran.

### 4.8. Histology

Colon tissues were fixed in 4% paraformaldehyde and then embedded in paraffin. Fixed tissues were cut into 3-μm-thick sections, placed on glass slides, and deparaffinized. The sections were stained with hematoxylin and eosin (H&E) following the standard protocol. Inflammation was blindly scored on the degree of inflammatory cell infiltration (from 0, normal, to 3, severe) and ulceration (from 0, normal, to 3, severe).

### 4.9. Statistical Analysis

Statistical analysis was performed using GraphPad Prism software. Statistical significance was evaluated with a two-tailed Student’s *t* test. All values are expressed as the mean ± SEM of data obtained from at least three independent experiments. Results were considered statistically significant at *p* < 0.05.

## Data Availability

Not applicable.

## Supplementary Materials

The following supporting information can be downloaded at: https://www.mdpi.com/1422-0067/23/10/5840/s1.

## Appendix A

Raw data of the independent Western blot experiments.
